# Percutaneous Nephrolithotomy vs Ureteroscopy for Kidney Stones in Children

**DOI:** 10.1001/jamanetworkopen.2025.16749

**Published:** 2025-06-20

**Authors:** Jonathan S. Ellison, David I. Chu, Caleb P. Nelson, W. Robert DeFoor, Justin Ziemba, Jing Huang, Xianqun Luan, Michael Kurtz, Christina B. Ching, Pankaj P. Dangle, Anthony J. Schaeffer, Renea Sturm, Wayland Wu, Christopher Bayne, Nicolas Fernandez, Michael E. Chua, Romano DeMarco, Pamela Ellsworth, Brian Augelli, Jing Bi-Karchin, Rebecca D. McCune, Seth Vatsky, Susan Back, Zi Wang, Hunter Beck, Anna Kurth, Laura Kurth, Annabelle Pleskoff, Christopher B. Forrest, Gregory E. Tasian, and the PKIDS Care Improvement Network, Kyle Rove, Scott Sparks, Eric Nelson, Bruce Schlomer, Aaron Krill, Ching Man Carmen Tong, Abby Taylor, Puneeta Ramachandra, Andrew Stec, Pasquale Casale, Douglas Coplen, Nicolette Janzen, Krystal Bagley, Michelle Denburg, Kimberley Dickinson, Rosemary Laberee, Matt Lorenzo, Antoine Selman-Fermin, Joana Dos Santos, Campbell Grant, Kate Kraft, Bhalaajee Meenakshi-Sundaram

**Affiliations:** 1 Department of Urology, Medical College of Wisconsin, Milwaukee; 2 Department of Surgery, Division of Urology, Ann & Robert H. Lurie Children’s Hospital of Chicago, Chicago, Illinois; 3 Department of Urology, Boston Children’s Hospital, Boston, Massachusetts; 4 Division of Urology, Cincinnati Children’s Hospital Medical Center, Cincinnati, Ohio; 5 Division of Urology, Department of Surgery, Perelman School of Medicine at the University of Pennsylvania, Philadelphia; 6 Department of Biostatistics, Epidemiology, and Informatics, Perelman School of Medicine at the University of Pennsylvania, Philadelphia; 7 PolicyLab, The Children’s Hospital of Philadelphia, Philadelphia, Pennsylvania; 8 Department of Pediatric Urology, Kidney and Urinary Tract Center, Nationwide Children’s Hospital, Columbus, Ohio; 9 Department of Pediatric Urology, Riley Hospital for Children, Indianapolis, Indiana; 10 Division of Urology, Department of Surgery, University of Utah, Salt Lake City; 11 Department of Pediatric Urology, Mattel Children’s Hospital at UCLA (University of California, Los Angeles), Los Angeles; 12 Department of Pediatric Urology, Bristol-Myers Squibb Children’s Hospital at Robert Wood Johnson University Hospital, New Brunswick, New Jersey; 13 Department of Pediatric Urology, Carilion Children’s Pediatric Urology, Roanoke, Virginia; 14 Division of Pediatric Urology, Seattle Children’s Hospital, Seattle, Washington; 15 Division of Urology, The Hospital for Sick Children, Toronto, Ontario, Canada; 16 Division of Pediatric Urology, Department of Urology, University of Florida, Gainesville; 17 Nemours Children’s Hospital and University of Central Florida College of Medicine, Orlando; 18 Division of Urology, Department of Surgery, The Children’s Hospital of Philadelphia, Philadelphia, Pennsylvania; 19 Department of Radiology, The Children’s Hospital of Philadelphia, Philadelphia, Pennsylvania; 20 Patient & Family Research Partners, The Children’s Hospital of Philadelphia, Philadelphia, Pennsylvania; 21 Department of Pediatrics, Applied Clinical Research Center, The Children’s Hospital of Philadelphia, Philadelphia, Pennsylvania; 22 Division of Urology, Children’s Hospital Colorado, Aurora; 23 Division of Urology, Children’s Hospital of Los Angeles, Los Angeles, California; 24 Departments of Surgery and Urology, Children’s Hospital of Richmond at VCU, Richmond, Virginia; 25 Division of Urology, Children’s National Hospital of Washington, DC, Washington, DC; 26 Division of Urology, Children’s Hospital of Alabama, Birmingham; 27 Division of Urology, Nemours A. I. duPont Hospital for Children–Wilmington, Wilmington, Delaware; 28 Division of Pediatric Urology, Nemours Children’s Health, Jacksonville, Florida; 29 Pediatric Urology, Nemours Children’s Health, Delaware Valley, Wilmington; 30 Division of Urology, St Louis Children’s Hospital, St Louis, Missouri; 31 Division of Urology, Texas Children’s Hospital, Houston; 32 Department of Urology, The Children’s Hospital of Philadelphia, Philadelphia, Pennsylvania; 33 Division of Nephrology, The Children’s Hospital of Philadelphia, Philadelphia, Pennsylvania; 34 Applied Clinical Research Center, The Children’s Hospital of Philadelphia, Philadelphia, Pennsylvania; 35 Research Administration, The Children’s Hospital of Philadelphia, Philadelphia, Pennsylvania; 36 Division of Urology, The Children’s Hospital of Philadelphia, Philadelphia, Pennsylvania; 37 Division of Urology, Toronto SickKids, Toronto, Ontario, Canada; 38 Division of Urology, University of Kentucky, Lexington; 39 University of Michigan, Ann Arbor; 40 University of Oklahoma Health Sciences Center, Oklahoma City

## Abstract

**Importance:**

Based on expert opinion, clinical guidelines recommend percutaneous nephrolithotomy or shockwave lithotripsy for children and adolescents with kidney stones 20 mm or larger, without mention of ureteroscopy as an alternative.

**Objective:**

To compare clinical and patient-reported outcomes for percutaneous nephrolithotomy vs ureteroscopy in children and adolescents with kidney and/or ureteral stones.

**Design, Setting, and Participants:**

This prospective cohort study was performed at 31 medical centers in the US and Canada. Participants included patients aged 8 to 21 years undergoing surgery for kidney and/or ureteral stones between March 16, 2020, and July 31, 2023.

**Exposures:**

Percutaneous nephrolithotomy vs ureteroscopy.

**Main Outcomes and Measures:**

Stone clearance assessed by ultrasonography 6 (±2) weeks postoperatively. Secondary outcomes included patient-reported outcomes 1 week after surgery.

**Results:**

The study enrolled 1039 eligible patients (median age, 15.6 [IQR, 12.5-17.3] years; 629 female [60.5%]; 40 Black [3.8%]; 128 Hispanic [12.3%]; and 792 White [76.2%]). One hundred twenty-six urologists performed percutaneous nephrolithotomy for 98 kidneys and/or ureters and ureteroscopy for 1069, including 36 undergoing percutaneous nephrolithotomy and 43 undergoing ureteroscopy for stones larger than 15 mm. Stone clearance was 67.2% (95% CI, 46.0%-88.4%) for percutaneous nephrolithotomy and 73.4% (95% CI, 69.4%-77.4%) for ureteroscopy, a difference that was not statistically significant (risk difference, −6.2%; 95% CI, −27.7% to 15.4%). For stones larger than 15 mm, stone clearance was 94.0% (95% CI, 83.3%-100%) for percutaneous nephrolithotomy and 55.0% (95% CI, 32.9%-77.1%) for ureteroscopy, a statistically significant difference (risk difference, 39.0%; 95% CI, 14.4%-63.5%). Compared with ureteroscopy, percutaneous nephrolithotomy had significantly lower pain intensity (T score difference, −5.42; 95% CI, −10.38 to −0.46), pain interference (T score difference, −5.88; 95% CI, −11.02 to −0.75), anxiety (T score difference, −5.74; 95% CI, −9.26 to −2.22), psychological stress experiences (T score difference, −7.90; 95% CI, −13.13 to −2.67), sleep disturbance (T score difference, −5.57; 95% CI, −8.56 to −2.58), and urinary symptoms (symptom score difference, −6.37; 95% CI, −11.71 to −1.03) 1 week after surgery.

**Conclusions and Relevance:**

Compared with ureteroscopy, percutaneous nephrolithotomy had similar stone clearance and better lived experiences for children and adolescents and was associated with greater stone clearance of kidney stones larger than 15 mm. A future adequately powered prospective clinical trial is needed to reaffirm these results.

## Key Points

**Question:**

Is percutaneous nephrolithotomy or ureteroscopy more successful in removing large kidney stones in children and adolescents?

**Findings:**

In this cohort study of 1039 children and adolescents who had surgery to remove kidney and ureteral stones at 31 medical centers in the US and Canada, stone clearance for percutaneous nephrolithotomy was 67% compared with 73% for ureteroscopy, a difference that was not statistically significant. Percutaneous nephrolithotomy was associated with significantly less postoperative pain intensity, pain interference, anxiety, and urinary symptoms.

**Meaning:**

These findings suggest that percutaneous nephrolithotomy provides similar stone clearance and better postoperative lived experiences in children and adolescents.

## Teaser

This cohort study compares the outcomes associated with use of percutaneous nephrolithotomy vs ureteroscopy for clearance of kidney stones in children and adolescents.

## Introduction

Children and adolescents constitute the fastest-growing population of patients with kidney stones, which may result in pain and/or require surgery.^1,2^ Larger kidney and ureteral stones typically require more complex interventions.^3^ Percutaneous nephrolithotomy directly accesses the kidney through a small incision to fragment and remove stones.^3^ Ureteroscopy entails passing an endoscope without incisions through the urethra and urinary collecting system to fragment and remove stones.

The American Urological Association guidelines for the surgical management of urinary stones recommend percutaneous nephrolithotomy or shockwave lithotripsy as equivalent treatments for pediatric patients with kidney stones 20 mm or larger, with no mention of ureteroscopy.^4^ These guidelines are supported only by expert opinion and do not reflect contemporary surgical treatment of large kidney stones in children and adolescents.^4,5^

The Pediatric Kidney Stone (PKIDS) Care Improvement Network is a collaborative research network of 31 North American medical centers. The PKIDS study, a prospective cohort study to compare the clinical effectiveness of interventions for children and adolescents with kidney and/or ureteral stones, arose from the desire of patients and caregivers to understand the impact of interventions on kidney stone removal and patients’ lived experiences. We hypothesized that percutaneous nephrolithotomy would clear large urinary stones more effectively than ureteroscopy.

## Methods

### Study Design

The PKIDS study was an investigator-initiated, prospective cohort study embedded in the clinical care of children and adolescents undergoing surgery for kidney and/or ureteral stone(s).^6^ This report followed Strengthening the Reporting of Observational Studies in Epidemiology (STROBE) guidelines for reporting observational cohort studies and followed a previously published protocol (eAppendix 2 in Supplement 1).^7^ The study included patients undergoing kidney stone surgery between March 16, 2020, and July 31, 2023, at 31 medical centers in 22 US states and 1 Canadian province that participate in the PKIDS Network (eFigure 1 in Supplement 1). The Children’s Hospital of Philadelphia (CHOP) served as the data coordinating center and central Institutional Review Board, which approved the study.^7^ All participants provided assent or written informed consent, with parents or guardians providing written informed consent for children 17 years or younger.

### Study Population

English- and Spanish-speaking patients aged 8 to 21 years undergoing surgery for kidney and/or ureteral stones were enrolled following informed consent and remunerated for participation and completion of outcome measures via the CHOP Participant Research Cards Program. The lower age limit reflects the availability of self-reported and validated Patient-Reported Outcomes Measurement Information System (PROMIS) questionnaires, thereby avoiding heterogeneity of parent-proxy reports.^8^ The upper age limit reflects the upper limit for pediatric care identified by the American Academy of Pediatrics.^9^ The only exclusion criterion was when obtaining informed consent would confer additional risk, such as a patient needing emergent surgery due to impending urosepsis. We considered the first surgical procedure for stone removal for enrolled patients to avoid patient-level clustering that might arise from repeated procedures for subsequent stone events within the study period. The study flowchart is shown in the Figure. Patients self-reported race and ethnicity, categorized as American Indian or Alaska Native, Asian, Black, Hispanic, non-Hispanic, White, multiracial, or other. These data were included in the study per our a priori data collection plan.

**Figure.**
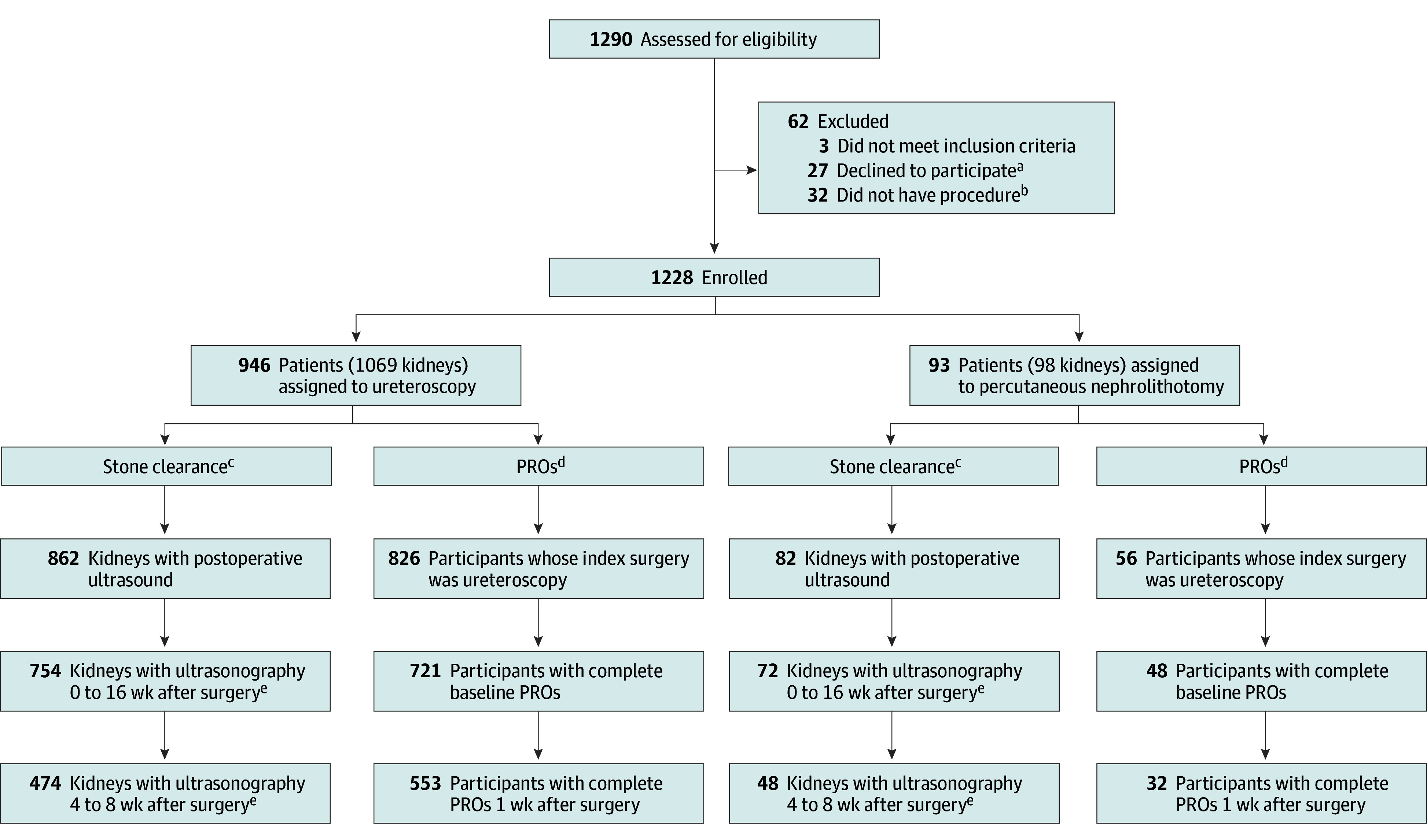
Study Flowchart The total enrolled 1228 patients include those with shockwave lithotripsy, a procedure that was not included in the reported current analysis. PRO indicates patient-reported outcome. ^a^Participants who withdrew but allowed data collection remained in the analysis cohort. ^b^Participants did not receive ureteroscopy, shockwave lithotripsy, or percutaneous nephrolithotomy. ^c^Treatment group allocation and PRO evaluation are at the patient level. ^d^Stone clearance was evaluated at the kidney level. ^e^Ultrasonographic studies reported either “no stone present” or “had measured stone size.”

### Patients and Caregivers as Collaborators in Study Design and Implementation

The PKIDS Patients and Family Partners are patients and caregivers included as members of the research team. These partners informed decisions regarding timing and choice of patient-reported outcomes (PROs), recruitment and retention efforts, and insight into reporting results.

### Outcomes

The primary outcome was stone clearance, defined as the absence of any stone larger than 4 mm assessed by renal ultrasonography at 6 weeks (±2 weeks) after index surgery using a standardized report template (eFigure 2 in Supplement 1). Stone clearance was measured at the kidney level, assuming independence of renal units for bilateral surgery. Ultrasonography eliminates ionizing radiation and is the preferred postoperative imaging for children. The 4-mm cut point for ultrasonographic-defined stone clearance acknowledges that ultrasonography typically overestimates size by approximately 2 mm,^10^ and 2 mm is a common definition for clinically insignificant stone fragments on computed tomography.^11^ A pediatric radiologist (S.B.) interpreted 10% of all preoperative and postoperative ultrasonography images and determined whether stone clearance was achieved after surgery. When the local and central reviews were discordant, a second pediatric radiologist (S.V.) reviewed images. The final determination of stone clearance was made by consensus between the central reviewers. Although the radiologists reading the ultrasonographic images obtained in clinical care were not blinded to the intervention, the central radiologists were blinded to intervention type.

Secondary outcomes were PROs of physical, emotional, and social health at 1 week after surgery. PKIDS Patient and Family Partners selected the following PROs to measure in the study: pain intensity (mean severity of pain), pain interference (degree to which pain interferes with daily functioning), anxiety, sleep disturbance, psychological stress experiences, peer relationships, and urinary symptoms.^8^ Pediatric PROMIS questionnaires with a 7-day recall for pain intensity, pain interference, anxiety, sleep disturbance, psychological stress experiences, and peer relationships were administered through Research Electronic Data Capture (REDCap) using computer-adaptive testing algorithms.^12,13^ Urinary symptoms were measured using the validated Dysfunctional Voiding Symptoms Score (range, 0-30, with higher scores indicating worse urinary symptoms)^14^ and a novel urinary symptom score, the Questionnaire for Urinary Issues–Kidney Stone Surgery (QUIKSS; range, 14-70, with higher scores indicating worse urinary symptoms) (eFigure 3 in Supplement 1). QUIKSS was developed by PKIDS investigators and the Patient and Family Partners using PROMIS methodology^15^ to measure symptoms experienced after kidney stone surgery, including symptoms (ie, hematuria) missing from other validated urinary symptom scores. We calibrated PROMIS scores to the T-score scale, with a mean of 50 and SD of 10 relative to the sample used for the instrument’s calibration.^16,17^ Raw scores were tabulated for urinary questionnaires.^14^

English- or Spanish-language questionnaires were administered before surgery (baseline) and postoperatively at 1 week (7 ± 3 days), 3 weeks (21 ± 7 days), 6 weeks (42 ± 7 days), and 3 months (90 ± 7 days). Emergency department visits, unplanned hospitalizations, surgical complications, surgical re-treatment, and self-reported days missed for school and/or work during the 3-month follow-up period were captured through patient self-report and medical record review.^14^

### Comparators

The comparators were percutaneous nephrolithotomy and ureteroscopy. Treatment was decided jointly among the operating urologist, patient, and/or caregiver. Each procedure, as well as variations such as stent placement, equipment used, or technical modifications, was performed at the discretion of the treating urologist. Shockwave lithotripsy was a potential treatment modality. Given its infrequent use, however, this analysis focused on percutaneous nephrolithotomy and ureteroscopy, reflecting contemporary management practices.

### Statistical Analysis

The a priori comparison in the published protocol and initial sample size calculations proposed to compare shockwave lithotripsy and percutaneous nephrolithotomy because guidelines recommend these procedures as first-line treatment for stones larger than 20 mm. A sample size of 294 patients undergoing shockwave lithotripsy and 92 patients undergoing percutaneous nephrolithotomy would have 80% power to detect an expected difference of 90%^18^ vs 65%^19^ in stone clearance between the procedures with a 2-sided α = .05, accounting for multiple comparisons and 15% loss to follow-up. However, the number of unbalanced confounders between these 2 groups and the few patients undergoing shockwave lithotripsy for calculi larger than 15 mm precluded meaningful comparisons. Therefore, we deviated from the predefined plan to compare ureteroscopy and percutaneous nephrolithotomy, reflecting clinical practice.

We developed propensity scores for treatment selection (eTable 1 in Supplement 1) using characteristics for participating surgeons (eTable 2 in Supplement 1) and medical centers (eTable 3 in Supplement 1) before study opening and of patients before surgery to ascertain potential confounders. We used inverse probability of treatment weighting to balance patient, surgeon, and medical center characteristics across treatment groups.^20^ We examined the balance of covariates using standardized mean differences before and after weighting, with a standardized mean difference of less than 0.10 after weighting indicating good balance^21,22,23^ (eFigure 4 in Supplement 1). Confounders with a standardized mean difference greater than 0.25 after weighting were included in the outcome models.

The primary analysis was based on intention to treat for the modality chosen at enrollment. We fit logistic regression models for stone clearance weighted with the inverse of the estimated propensity score, adding covariates that remained unbalanced after weighting. We used linear regression to estimate the association between surgical treatment and PROs 1 week after surgery, adjusting for baseline values. We defined minimal important change as 3 points for PROMIS measures.^24^ Additionally, the proportion of patients exceeding 50% of the SDs of the baseline PROs at 1 week in either direction were calculated for each treatment group, reflecting an alternative definition of minimally important change.^25^

We performed subgroup analyses to determine heterogeneity of treatment effect (HTE), which were exploratory and not powered to detect prespecified differences.^26^ For stone clearance, we assessed HTE for stone size (<7, 7 to 10, >10 to 15, and >15 mm) and location (ureter or ureteropelvic junction, non–lower pole kidney, and lower pole kidney). For PROs, we examined HTE by sex and age (8-11, 12-15, 16-18, or 19-21 years). These categories were determined a priori considering input from PKIDS urologists before finalizing the statistical analysis plan (eAppendix 1 in Supplement 1).

Enrollment began in March 2020, coinciding with the COVID-19 pandemic, which deferred much routine in-person care, including postoperative imaging. We therefore performed sensitivity analyses. First, we performed multiple imputation for missing data with different random and nonrandom assumptions for missing data as a sensitivity analysis. We also reported characteristics of the entire enrolled and analyzed cohorts for patients undergoing ureteroscopy and percutaneous nephrolithotomy in eTable 4 in Supplement 1. Second, we analyzed stone clearance by considering an expanded ascertainment window for ultrasonographic images obtained until 16 weeks after surgery using logistic regression models for stone clearance weighted with the inverse of the estimated propensity score. Third, we assessed stone clearance by the proportion of centrally reviewed misclassified stone clearance. To evaluate generalizability, we compared demographic characteristics and types of surgery of study participants with patients aged 8 to 21 years who had kidney stone surgery during the same time period at 58 institutions within the National Patient-Centered Clinical Research Network (PCORnet).

A 2-sided *P* = .05 was the threshold for statistical significance. Statistical analyses were conducted using R statistical software, version 4.4 (R Program for Statistical Computing).

## Results

For the primary analysis, 1039 patients with a median age of 15.6 (IQR, 12.5-17.3) years and median largest stone size of 6.0 (IQR, 4.0-9.6) mm were included. In total, 629 patients (60.5%) were female and 410 (39.5%) were male. In terms of race, 3 patients (0.3%) were American Indian or Alaska Native; 15 (1.4%), Asian; 40 (3.8%), Black; 792 (76.2%), White; 50 (4.8%), multiracial; 34 (3.3%), other; and 105 (10.1%), unknown. A total of 128 patients (12.3%) were Hispanic. One hundred twenty-six urologists treated 98 kidneys and/or ureters with percutaneous nephrolithotomy and 1069 with ureteroscopy (Figure). Table 1 shows the characteristics of patients undergoing percutaneous nephrolithotomy and ureteroscopy. eTable 5 in Supplement 1 shows characteristics of patients undergoing shockwave lithotripsy. eFigure 4 in Supplement 1 displays the balance of preoperative characteristics before and after propensity score weighting. Thirty-six patients underwent percutaneous nephrolithotomy and 43 underwent ureteroscopy for large stones (>15 mm). Ureteral stents were placed in 45 of 97 children (46.4%) undergoing percutaneous nephrolithotomy and 841 of 1064 (79.0%) undergoing ureteroscopy, respectively. Median stent dwell times were 17 (IQR, 5-47) days for percutaneous nephrolithotomy and 5 (IQR, 4-14) days for ureteroscopy. eTable 6 in Supplement 1 displays other surgical characteristics (ie, energy source and tract size). Five hundred and twelve of 1046 participants (49.0%) completed postoperative imaging at the prespecified 4-week imaging window, while 827 of 1046 (79.1)% completed ultrasonography by 16 weeks after surgery.

**Table 1.** Characteristics of Patients Who Underwent PCNL and URS

Characteristic	Treatment group		
	PCNL (n = 93)	URS (n = 946)	Overall (N = 1039)^a^
Age, median (IQR), y	15.0 (12.0-17.3)	15.6 (12.6-17.3)	15.65 (12.5-17.3)
Age group, No. (%), y			
8-11	24 (25.8)	190 (20.1)	214 (20.6)
12-15	32 (34.4)	336 (35.5)	368 (35.4)
16-18	22 (23.7)	337 (35.6)	359 (34.6)
19-21	15 (16.1)	83 (8.8)	98 (9.4)
Sex, No. (%)			
Female	43 (46.2)	586 (61.9)	629 (60.5)
Male	50 (53.8)	360 (38.1)	410 (39.5)
Race, No. (%)			
American Indian or Alaska Native	1 (1.1)	2 (0.2)	3 (0.3)
Asian	1 (1.1)	14 (1.5)	15 (1.4)
Black	2 (2.2)	38 (4.0)	40 (3.8)
White	58 (62.4)	734 (77.6)	792 (76.2)
Multiracial	4 (4.3)	46 (4.9)	50 (4.8)
Other (not specified)	7 (7.5)	27 (2.9)	34 (3.3)
Missing or unknown	20 (21.5)	85 (9.0)	105 (10.1)
Ethnicity, No. (%)			
Hispanic	18 (19.4)	110 (11.6)	128 (12.3)
Non-Hispanic	58 (62.4)	746 (78.9)	804 (77.4)
Missing or unknown	17 (18.3)	90 (9.5)	107 (10.3)
BMI, median (IQR)	20.4 (16.9-26.2)	22.2 (18.8-27.7)	22.1 (18.6-27.6)
No. of stones in treated kidney, median (IQR)	2.0 (1.0-3.0)	1.0 (1.0-2.0)	1.0 (1.0-2.0)
Total stone size in treated kidney, median (IQR), mm	19.0 (11.0-27.0)	7.0 (4.0-11.0)	7.0 (4.0-12.0)
Largest stone size in treated kidney, median (IQR), mm	15.0 (9.0-20.0)	6.0 (4.0-9.0)	6.0 (4.0-9.6)
Largest stone size, No. (%), mm			
No stone present	1 (1.1)	55 (5.8)	56 (5.4)
<7	15 (16.1)	447 (47.3)	462 (44.5)
7-10	9 (9.7)	246 (26.0)	255 (24.5)
11-15	20 (21.5)	100 (10.6)	120 (11.5)
>15	36 (38.7)	43 (4.5)	79 (7.6)
Missing size	11 (11.8)	41 (4.3)	52 (5.0)
Missing or unknown	1 (1.1)	14 (1.5)	15 (1.4)
Stone location, No. (%)			
No stone	1 (1.1)	55 (5.8)	56 (5.4)
Lower pole kidney	25 (26.9)	164 (17.3)	189 (18.2)
Non–lower pole kidney	53 (57.0)	256 (27.1)	309 (29.7)
Ureter (includes UPJ)	13 (14.0)	457 (48.3)	470 (45.2)
Missing or unknown	1 (1.1)	14 (1.5)	15 (1.4)
Prior stone surgery, No. (%)	23 (24.7)	160 (16.9)	183 (17.6)
Primary indication for surgery, No. (%)			
Elective	4 (4.3)	97 (10.3)	101 (9.7)
Pain	27 (29.0)	634 (67.0)	661 (63.6)
UTI	54 (58.1)	172 (18.2)	226 (21.8)
Other (not specified)	8 (8.6)	43 (4.5)	51 (4.9)
Presurgical drainage, No. (%)			
None	58 (62.4)	655 (69.2)	713 (68.6)
Stent	11 (11.8)	267 (28.2)	278 (26.8)
Nephrostomy	21 (22.6)	10 (1.1)	31 (3.0)
Other (not specified)	2 (2.2)	3 (0.3)	5 (0.5)
Missing or unknown	1 (1.1)	11 (1.2)	12 (1.2)
Prepresentation ED visit, No. (%)	39 (41.9)	641 (67.8)	680 (65.4)
Structural renal abnormality in treated side, No. (%)			
Any abnormality	21 (22.6)	112 (11.8)	133 (12.8)
Horseshoe kidney	2 (2.2)	3 (0.3)	5 (0.5)
Malrotation	7 (7.5)	7 (0.7)	14 (1.3)
Pelvic kidney	3 (3.2)	5 (0.5)	8 (0.8)
Chronic hydronephrosis	5 (5.4)	25 (2.6)	30 (2.9)
Cross-fused extopia	0	1 (0.1)	1 (0.1)
Complete duplication	0	1 (0.1)	1 (0.1)
Partial duplication	1 (1.1)	13 (1.4)	14 (1.3)
Transplant kidney	2 (2.2)	3 (0.3)	5 (0.5)
Calyceal diverticulum	0	23 (2.4)	23 (2.2)
Other (not specified)	3 (3.2)	37 (3.9)	40 (3.8)
Missing or unknown	2 (2.2)	8 (0.8)	10 (1.0)
Comorbid conditions, No. (%)			
Neurogenic bladder	17 (18.3)	78 (8.2)	95 (9.1)
Ventilator dependent	6 (6.5)	11 (1.2)	17 (1.6)
Neuromuscular disorder	33 (35.5)	116 (12.3)	149 (14.3)
Hematologic disorder	9 (9.7)	42 (4.4)	51 (4.9)
Oxygen support	9 (9.7)	25 (2.6)	34 (3.3)
Cardiac risk factors	5 (5.4)	32 (3.4)	37 (3.6)
Structural CNS abnormality	4 (4.3)	26 (2.7)	30 (2.9)
Developmental delay	37 (39.8)	113 (11.9)	150 (14.4)
Epilepsy	21 (22.6)	65 (6.9)	86 (8.3)
Food insecurity, No. (%)			
Often true	4 (4.3)	9 (1.0)	13 (1.3)
Sometimes true	4 (4.3)	59 (6.2)	63 (6.1)
Never true	64 (68.8)	761 (80.4)	825 (79.4)
Missing or unknown	21 (22.6)	117 (12.4)	138 (13.3)

Abbreviations: BMI, body mass index (calculated as the weight in kilograms divided by the height in meters squared); CNS, central nervous system; ED, emergency department; PCNL, percutaneous nephrolithotomy; UPJ, ureteropelvic junction; URS, ureteroscopy; UTI, urinary tract infection.

^a^ The characteristics of 7 patients who had PCNL with contralateral URS are described in the PCNL column.

Study participants were younger than nonstudy patients who had kidney stone surgery at PKIDS sites and other institutions within PCORnet. The study sample had statistically significant lower proportions of Hispanic patients and members of racial minority groups than PCORnet populations at non-PKIDS sites, although differences were small (eFigures 5-8 in Supplement 1). Using the pediatric medical complexity algorithm,^27^ participants undergoing percutaneous nephrolithotomy were more medically complex than at other PCORnet institutions (eTable 7 in Supplement 1).

### Stone Clearance

Ultrasonography completion is shown in the Figure. For the primary analysis, stone clearance was 67.2% (95% CI, 46.0%-88.4%) for percutaneous nephrolithotomy and 73.4% (95% CI, 69.4%-77.4%) for ureteroscopy, a difference that was not statistically significant (risk difference, −6.2%; 95% CI, −27.7% to 15.4%). Logistic regression models for stone clearance including unbalanced covariates following propensity score weighting also found no difference in stone clearance (odds ratio, 1.11; 95% CI, 0.31-3.98). In the centralized imaging review (258 participants), the sensitivity and specificity of local ultrasonographic interpretation were 97% and 79%, respectively, which were similar between treatment groups.

In HTE analyses for stones larger than 15 mm, stone clearance for percutaneous nephrolithotomy was 94.0% (95% CI, 83.3%-100%) compared with 55.0% (95% CI, 32.9%-77.1%) for ureteroscopy (risk difference, 39.0% [95% CI, 14.4%-63.5%]; *P* = .002) (Table 2). No differences were observed between treatments for stones 15 mm or smaller or by location. eFigure 9 in Supplement 1 shows stone clearance for percutaneous nephrolithotomy and ureteroscopy with consideration of preoperative stone size as a continuous variable.

**Table 2.** Stone Clearance for PCNL and URS as an Overall Weighted Comparison and for Heterogeneity of Treatment Effect by Stone Size and Stone Location

Covariate^a^	Weighted PCNL vs URS		
	PCNL group	URS group	Risk difference between PCNL and URS
Stone clearance, % (95% CI)	67.2 (46.0 to 88.4)	73.4 (69.4 to 77.4)	−6.2 (−27.7 to 15.4)
Stone size, % (95% CI), mm			
<7	52.5 (0 to 100)	80.5 (75.1 to 85.8)	−28.0 (−83.8 to 27.9)
7-10	46.5 (0 to 100)	67.4 (59.4 to 75.4)	−20.9 (−90.6 to 48.8)
11-15	68.7 (34.6 to 100)	62.7 (49.4 to 76.1)	6.0 (−30.7 to 42.6)
>15	94.0 (83.3 to 100)	55.0 (32.9 to 77.1)	39.0 (14.4 to 63.5)
Stone location, % (95% CI)			
Lower pole kidney	47.6 (3.8 to 91.5)	64.1 (54.3 to 74.0)	−16.5 (−61.4 to 28.4)
Non–lower pole kidney	71.2 (43.2 to 99.3)	58.6 (49.6 to 67.6)	12.6 (−16.9 to 42.1)
Ureter^b^	75.3 (33.8 to 100)	83.3 (78.4 to 88.2)	−8.0 (−49.8 to 33.7)

Abbreviations: PCNL, percutaneous nephrolithotomy; URS, ureteroscopy.

^a^ Covariates that remained unbalanced following propensity score matching include age group, monogenic stone disease, presence of neuromuscular comorbidity, presence of structural renal anomaly, primary indication for surgery, surgery performed within 24 hours of symptom onset, restrictions to operating room at institution, type of preoperative imaging, number of kidney stones visualized, stone location, and maximum of longest stone dimension.

^b^ Includes stones at the ureteropelvic junction.

### Patient Experiences

Compared with ureteroscopy, patients undergoing percutaneous nephrolithotomy reported lower pain intensity (T score difference, −5.42; 95% CI, −10.38 to −0.46), pain interference (T score difference, −5.88; 95% CI, −11.02 to −0.75), anxiety (T score difference, −5.74; 95% CI, −9.26 to −2.22), stress (T score difference, −7.90; 95% CI, −13.13 to −2.67), sleep disturbance (T score difference, −5.57; 95% CI, −8.56 to −2.58), and urinary symptoms (symptom score difference, −6.37; 95% CI, −11.71 to −1.03) 1 week after surgery (Table 3). More patients in the ureteroscopy group compared with the percutaneous nephrolithotomy group exceeded at least a 50% change in the baseline SD of PRO scores for urinary symptoms as measured by QUIKSS (42.7% [95% CI, 38.5%-46.8%] vs 21.9% [95% CI, 3.3%-40.5%]), sleep disturbance (30.6% [95% CI, 26.8%-34.3%] vs 10.1% [95% CI, 0%-24.2%]), and stress experiences (22.6% [95% CI, 19.2%-25.9%] vs 3.6% [95% CI, 0%-7.8%]) 1 week after surgery (eTable 8 in Supplement 1). In HTE analyses, female patients had statistically significant lower procedure effect scores for urinary symptom scores (−7.47 [95% CI, −12.86 to −2.08]), anxiety (−7.43 [95% CI, −11.78 to −3.09]), pain interference (−7.26 [95% CI, −14.43 to −0.09]), sleep disturbance (−5.58 [95% CI, −9.77 to −1.39]), and stress experience (−11.19 [95% CI, −17.11 to −5.27]) 1 week after percutaneous nephrolithotomy compared with ureteroscopy (eTable 9 in Supplement 1). There were no consistent patterns in HTE analyses by age groups for percutaneous nephrolithotomy compared with ureteroscopy (eTable 10 in Supplement 1).

**Table 3.** Association of Study Treatment With PROs of Physical, Emotional, and Social Health at 1 Week After Surgery

PRO instrument	Treatment group, weighted mean score (95% CI)				Preoperative-postoperative differences		Procedure effect of PCNL compared with URS, controlling for baseline
	Baseline		Postoperative		PCNL	URS	
	PCNL (n = 48)	URS (n = 721)	PCNL (n = 32)	URS (n = 553)			
PROMIS Anxiety^a^	51.32 (45.42 to 57.22)	51.19 (50.33 to 52.04)	45.89 (41.15 to 50.62)	50.32 (49.46 to 51.17)	−6.64 (−11.08 to −2.21)	−0.85 (−1.62 to −0.08)	−5.74 (−9.26 to −2.22)
PROMIS Pain Intensity^a^	42.51 (38.47 to 46.55)	48.31 (47.54 to 49.08)	42.72 (37.68 to 47.76)	49.23 (48.61 to 49.84)	−0.32 (−5.44 to 4.81)	0.79 (−0.05 to 1.64)	−5.42 (−10.38 to −0.46)
PROMIS Pain Interference^a^	49.27 (43.34 to 55.19)	55.27 (54.34 to 56.20)	52.43 (47.34 to 57.53)	58.64 (57.88 to 59.40)	2.15 (−5.02 to 9.32)	3.49 (2.47 to 4.50)	−5.88 (−11.02 to −0.75)
PROMIS Peer Relationships^a^	46.32 (43.19 to 49.44)	47.78 (47.10 to 48.46)	47.87 (44.26 to 51.48)	47.43 (46.75 to 48.11)	1.92 (−1.90 to 5.75)	−0.19 (−0.78 to 0.40)	1.63 (−1.94 to 5.20)
PROMIS Sleep Disturbances^a^	55.25 (51.54 to 58.97)	57.25 (56.42 to 58.09)	51.73 (47.88 to 55.59)	57.7 (56.91 to 58.49)	−4.16 (−7.04 to −1.28)	0.47 (−0.35 to 1.28)	−5.57 (−8.56 to −2.58)
PROMIS Stress Experiences^a^	55.34 (51.53 to 59.16)	54.32 (53.53 to 55.12)	47.74 (42.47 to 53.00)	53.89 (53.10 to 54.69)	−8.57 (−14.14 to −3.00)	−0.32 (−1.01 to 0.36)	−7.9 (−13.13 to −2.67)
DVSS score^b^	4.86 (3.37 to 6.35)	6.93 (6.59 to 7.27)	7.63 (4.99 to 10.26)	7.77 (7.40 to 8.15)	2.37 (0.02 to 4.71)	0.75 (0.41 to 1.09)	0.91 (−1.49 to 3.31)
QUIKSS score^c^	9.37 (4.13 to 14.61)	14.77 (13.85 to 15.70)	11.48 (6.24 to 16.72)	19.52 (18.63 to 20.40)	1.87 (−4.64 to 8.38)	4.49 (3.4 to 5.58)	−6.37 (−11.71 to −1.03)

Abbreviations: DVSS, Dysfunctional Voiding Symptom Score; PCNL, percutaneous nephrolithotomy; PROMIS, Patient-Reported Outcomes Measurement Information System; QUIKSS, Questionnaire for Urinary Issues–Kidney Stone Surgery; URS, ureteroscopy.

^a^ T scores range from 0 to 100 with higher T scores indicating worse patient-reported outcomes for pain intensity, pain interference, anxiety, sleep disturbances, and stress experiences and better patient-reported outcomes for peer relationships.

^b^ Scores range from 0 to 30, with higher scores indicating worse urinary symptoms.

^c^ Consists of 16 items that used a frequency response scale and a 7-day recall period. Each item was scored from never (0) to always (4). The total score was the sum of the items. Scores range from 0 to 56.

There were no statistically significant differences in unanticipated health care visits or surgical complications. A small yet statistically significant higher frequency of unanticipated secondary interventions was seen in the ureteroscopy group compared with the percutaneous nephrolithotomy group (risk difference, −3.6; 95% CI, −4.8 to −2.4) (Table 4).

**Table 4.** Frequency of Unanticipated Postoperative Health Care Encounters After PCNL and URS

Unanticipated postoperative health care encounters	Weighted PCNL vs URS, % (95% CI)		
	PCNL group	URS group	Risk difference between groups
Unexpected acute health visits	21.1 (7.2 to 35.0)	14.4 (12.1 to 16.6)	6.7 (−7.4 to 20.8)
ED or urgent care visits	7.3 (0 to 14.7)	10.3 (8.3 to 12.2)	−2.9 (−10.6 to 4.7)
Inpatient admissions	17.9 (4.4 to 31.4)	6.9 (5.3 to 8.5)	11.0 (−2.5 to 24.6)
Other acute visits	1.6 (0 to 4.8)	1.0 (0.3 to 1.6)	0.7 (−2.6 to 3.9)
Unanticipated procedure	0 (0 to 0)	3.6 (2.4 to 4.8)	−3.6 (−4.8 to −2.4)

Abbreviations: ED, emergency department; PCNL, percutaneous nephrolithotomy; URS, ureteroscopy.

### Sensitivity Analyses

Sensitivity analyses evaluating ultrasonographic images obtained 0 to 16 weeks after surgery (eTable 11 in Supplement 1) and accounting for misclassification of stone clearance in local ultrasonographic interpretations (eTable 12 in Supplement 1) were similar to the primary analysis. In multiple imputation analyses, the difference in stone clearance between percutaneous nephrolithotomy and ureteroscopy for stones larger than 15 mm was no longer statistically significant (eTable 13 in Supplement 1). Results for the analyses of PROs using imputed data were similar to those of the complete data analyses.

## Discussion

In this multicenter prospective cohort study that compared percutaneous nephrolithotomy and ureteroscopy for children and adolescents with kidney and/or ureteral calculi, there were no differences in stone clearance, but patient experiences were better for percutaneous nephrolithotomy. In a priori heterogeneity of treatment effect analyses, children and adolescents undergoing treatment for large calculi (>15 mm) with percutaneous nephrolithotomy had better stone clearance than those undergoing ureteroscopy. Shockwave lithotripsy was rarely used for large stones at 31 medical centers in North America, despite American Urological Association guidelines supporting shockwave lithotripsy as a first-line option for large kidney stones.^4^ These results strengthen the evidence base supporting percutaneous nephrolithotomy as the first-line surgical intervention for pediatric patients with kidney stones larger than 15 mm and underscore the low contemporary use of shockwave lithotripsy for large kidney stones.

Current US guidelines recommend percutaneous nephrolithotomy and shockwave lithotripsy as first-line surgical treatment for pediatric patients with large kidney stones based on expert opinion.^28^ Although we initially proposed to compare shockwave lithotripsy and percutaneous nephrolithotomy to align with clinical guidelines, only 5 patients underwent shockwave lithotripsy for stones larger than 15 mm. This supports our finding of surgeon preferences, wherein many urologists across the US and Canada did not consider shockwave lithotripsy a viable option for large stones, emphasizing the lack of clinical equipoise for these treatments.^5^ Instead, urologists considered either percutaneous nephrolithotomy or ureteroscopy, despite limited evidence to support decisions between these treatment modalities. Prior studies that compared alternative treatments of large kidney stones in children and adolescents^29,30,31^ are limited by retrospective design, heterogenous outcome definitions, and/or lack of generalizability. Our study addresses many of these uncertainties, further supporting greater stone clearance for percutaneous nephrolithotomy compared with ureteroscopy for stones larger than 15 mm.

Clearance rates for percutaneous nephrolithotomy for large stones from this study are consistent with rates exceeding 90% from previous studies.^32,33,34^ However, similar studies of ureteroscopy for larger stones often report stone clearance rates higher than 55%.^33,34^ These differences, namely the underperformance of ureteroscopy in the PKIDS study for larger stones, could reflect the use of standardized imaging assessments and the clinical experience of the 126 urologists across 31 hospitals, representing a diversity of health care delivery and surgeon experience not typical of prior studies.

Nonetheless, uncertainty remains regarding stone clearance for percutaneous nephrolithotomy compared with ureteroscopy. Differences in stone clearance lost statistical significance following imputation. Notably, stone clearance rates were seemingly lower for stones 15 mm or smaller than those larger than 15 mm in the percutaneously nephrolithotomy group, suggesting confounding due to patient and/or stone complexity beyond stone size. These results should be confirmed in future prospective trials specifically powered to detect differences between treatment modalities for stones larger than 15 mm. Notably, our results were robust to other sensitivity analyses, such as those considering an expanded outcome ascertainment window thereby reducing missingness.

Our study includes the largest analysis, to our knowledge, of patient experiences using validated measures after kidney stone surgery. Unexpectedly, patients reported less pain interference, pain intensity, anxiety, sleep disturbances, and urinary symptoms following percutaneous nephrolithotomy compared with ureteroscopy, with each significant difference in PROMIS T scores exceeding 3 points, a commonly used threshold for minimal important change.^24^ As minimal important change values for PROMIS measures have not been described in the pediatric population with kidney stones, we evaluated an alternate indicator of minimally important change, with more patients receiving ureteroscopy reporting increases in urinary, stress, and sleep disturbance scores that exceeded 50% of the SD of baseline scores.^25^ The better experiences with percutaneous nephrolithotomy may be explained by the lower use of ureteral stents and ureteral manipulation for this procedure. Further investigation is needed to understand whether PRO differences reported herein are primarily related to ureteral stenting or to ureteroscopy, irrespective of stent use.^35^

### Limitations

Limitations of the study include lack of randomization, owing to the strong preferences for particular surgical interventions among urologists and patients and the resulting lack of clinical equipoise.^5^ Therefore, we designed this study as an observational study while mitigating confounding and bias through measuring and balancing granular patient-, surgeon-, and medical center–level variables across treatment groups. Second, postoperative follow-up of participants was impacted by the COVID-19 pandemic, which began at the time the first patient was enrolled, with 49.0% of participants completing postoperative imaging at the prespecified imaging window. However, 79.1% of patients completed ultrasonography by 16 weeks after surgery, which was higher than follow-up imaging rates in a large claims database.^36,37^ Finally, we did not consider technical aspects of surgery such as access tract size, patient positioning, access and exit strategies, and energy sources. Analyses of these technical factors could provide surgeons with information to improve percutaneous nephrolithotomy outcomes.

## Conclusions

In this cohort study, percutaneous nephrolithotomy was associated with greater clearance of large kidney stones and better physical, social, and emotional health for children and adolescents after surgery compared with ureteroscopy. Shockwave lithotripsy is not commonly used for children and adolescents with kidney stones larger than 15 mm. A future adequately powered prospective clinical trial is needed to reaffirm these results.

## Notes

Supplement 1. **eAppendix 1.** Statistical Analysis Plan **eTable 1.** Final List of Confounders Used in the Propensity Score Model **eFigure 1.** The Pediatric KIDney Stone (PKIDS) Care Improvement Network **eFigure 2.** Ultrasonographic Protocol Agreed Upon by PKIDS Investigators and Implemented as Standard Imaging Follow-Up After Surgical Intervention **eFigure 3.** Questionnaire for Urinary Issues Following Kidney Stone Surgery **eFigure 4.** Balance of Preoperative Characteristics Before and After Propensity Score Weighting **eFigure 5.** Distribution of age of PKIDS Trial Participants and at PCORnet Sites That Participate in PKIDS and PCORnet Sites That Do Not Participate in PKIDS **eFigure 6.** Distribution of Sex of PKIDS Trial Participants and at PCORnet Sites That Participate in PKIDS and PCORnet Sites That Do Not Participate in PKIDS **eFigure 7.** Distribution of Race of PKIDS Trial Participants and at PCORnet Sites That Participate in PKIDS and PCORnet Sites That Do Not Participate in PKIDS **eFigure 8.** Distribution of Ethnicity of PKIDS Trial Participants and at PCORnet Sites That Participate in PKIDS and PCORnet Sites That Do Not Participate in PKIDS **eFigure 9.** Difference in Stone Clearance Between Percutaneous Nephrolithotomy (PCNL) Compared With Ureteroscopy (URS) With Preoperative Stone Size Analyzed as a Continuous Variable **eTable 2.** Characteristics of Urologists Participating in the PKIDS Trial **eTable 3.** Characteristics of Medical Centers Participating in the PKIDS Trial **eTable 4.** Characteristics of the Enrolled and Primary Analytic Cohorts of Patients Who Underwent Percutaneous Nephrolithotomy (PCNL) and Ureteroscopy (URS) **eTable 5.** Characteristics of Patients Who Underwent Shockwave Lithotripsy (SWL) **eTable 6.** Characterization of Energy Sources and Tract Size for Patients Undergoing Percutaneous Nephrolithotomy (PCNL) and Ureteroscopy (URS) **eTable 7.** Distribution of Sex of PKIDS Trial Participants at PCORnet Sites That Participate in PKIDS and PCORnet Sites That Do Not Participate in PKIDS **eTable 8.** Proportion of Participants Undergoing Percutaneous Nephrolithotomy (PCNL) and Ureteroscopy (URS) Whose Difference in Score at 1 Week Exceeded 30% and 50% of the SD of the Patient-Reported Outcome Measure Among All Participants at Baseline **eTable 9.** Impact of Percutaneous Nephrolithotomy (PCNL) and Ureteroscopy (URS) on Patient-Reported Outcomes (PRO), by Sex, at 1 Week Following Intervention **eTable 10.** Impact of Percutaneous Nephrolithotomy (PCNL) and Ureteroscopy (URS) on Patient-Reported Outcomes (PRO), by Age Group, at 1 Week Following Intervention **eTable 11.** Stone Clearance for Percutaneous Nephrolithotomy (PCNL) and Ureteroscopy (URS) Considering Ultrasonographic Images Obtained From 0 to 16 Weeks After Surgery, Overall and by Stone Size and Stone Location **eTable 12.** Stone Clearance for Percutaneous Nephrolithotomy (PCNL) and Ureteroscopy (URS) Considering Sensitivity and Specificity of Ultrasonographic Images on Centralized Imaging Review, Overall and by Stone Size and Stone Location **eTable 13.** Stone Clearance for Percutaneous Nephrolithotomy (PCNL) and Ureteroscopy (URS) Based on Multiple Imputation for Missing Ultrasonographic Data at the 4- to 8-Week Interval, Overall and by Stone Size and Stone Location **eAppendix 2.** Study Protocol

Supplement 2.
Data Sharing Statement
