# Supplementary material for: Percutaneous Nephrolithotomy vs Ureteroscopy for Kidney Stones in Children
Source: JAMA Netw Open. 2025 Jun 20;8(6):e2516749. doi: 10.1001/jamanetworkopen.2025.16749 (PMC12181786; doi:10.1001/jamanetworkopen.2025.16749)
Supplement: Supplement 2. — Data Sharing Statement [file jamanetwopen-e2516749-s002.pdf]

Data Sharing Statement

Ellison. Percutaneous Nephrolithotomy vs Ureteroscopy for Kidney Stones in Children. *JAMA Netw Open*. Published June 20, 2025. doi:10.1001/jamanetworkopen.2025.16749

Data
Additional Information: ClinicalTrials.gov Identifier: NCT04285658
Data available: Yes
Data types: Deidentified participant data, Data dictionary
How to access data: Investigators may submit proposals for secondary analysis of PKIDS trial data, which will be reviewed by the PKIDS Publications and Study Initiation Committee per the Publications Guidelines. Proposals for data access will be considered from investigators in and outside the PKIDS network.
When available: beginning date: 10-10-2025

Supporting Documents
Document types: Statistical/analytic code, Informed consent form
How to access documents: Please request statistical analysis code and ICFs from Dr. Ellison at jellison@childrenswi.org
When available: With publication

Additional Information
Who can access the data: Researchers whose proposed use of the data has been approved
Types of analyses: Analyses that align with the mission of the Pediatric KIDney Stone (PKIDS) Care Improvement Network, which is to improve the health of children with kidney stone disease.
Mechanisms of data availability: After approval of a proposal and the necessary data use agreements.
